# Prenatal Factors Influencing Calf Morbidity and Mortality in Dairy Cattle: A Systematic Review of the Literature (2000–2024)

**DOI:** 10.3390/ani15121772

**Published:** 2025-06-16

**Authors:** Lukas Trzebiatowski, Frederike Wehrle, Markus Freick, Karsten Donat, Axel Wehrend

**Affiliations:** 1 Veterinary Clinic for Reproductive Medicine and Neonatology, Justus-Liebig University Giessen, 35392 Giessen, Germany; kdonat@thtsk.de (K.D.); axel.wehrend@vetmed.uni-giessen.de (A.W.); 2 ZAFT e.V., Centre for Applied Research and Technology, HTW Dresden—University of Applied Sciences, 01069 Dresden, Germany; frederike.wehrle@gmx.de; 3 Institute of Agricultural and Nutritional Sciences, Martin Luther University Halle-Wittenberg, 06120 Halle (Saale), Germany; markus.freick@landw.uni-halle.de; 4 Thuringian Animal Disease Fund (Institution by Law, Animal Health Service, Thüringer Tierseuchenkasse AdöR), 07745 Jena, Germany

**Keywords:** gestation, epigenetic effects, calf welfare

## Abstract

This study aimed to systematically review the literature of the last 24 years to determine the influence of dam heat stress, nutrition, body condition, vaccination, parity, and twin pregnancy on calf morbidity and mortality. The systematic search was carried out using PubMed, CAB Abstracts, and Web of Science databases. The final number of included studies was 11 for heat stress, 21 for nutrition, 11 for body condition, 11 for vaccination, 23 for parity, and 18 for twin pregnancy. The body condition score, parity, and twin pregnancy had an influence on perinatal mortality. Vaccination, parity, and twin pregnancy had an influence on mortality up to weaning. Heat stress, nutrition, and twin pregnancy had an influence on the immunoglobulin transfer to the calves. Nutrition, body condition score, vaccination, and parity had an influence on morbidity. This systematic review provides evidence that prenatal factors have an influence on calf morbidity and mortality.

## Simple Summary

Despite advances in calf husbandry, poor calf health and increased calf mortality are problems faced on farms. This systematic review aimed to assess the influence of various antepartum factors on calf morbidity and mortality. This will help set the course for the rearing of healthy and productive calves even before birth and thus counteract the public perception of unsatisfactory calf health.

## 1. Introduction

Calf health has a direct effect on the profitability and productivity of dairy farms. On the one hand, it is linked to the costs of treating diseases and the loss of animals and, on the other hand, linked to reduced future performance of the replacement stock [1,2,3]. In addition, calf mortality is an indicator of animal welfare [4,5]. Lower rates of calves with insufficient transmission of immunoglobulins reduce calf morbidity and mortality and, therefore, are an indicator of improved calf welfare [6]. Through management, preventative measures, and appropriate feeding, good calf health can be achieved, and calves can reach their full potential for growth and performance [7]. Antepartum, intrapartum, and postpartum factors all have an influence on calf mortality and morbidity [8]. Reported perinatal mortality ranges from 2 to 10% [9,10]. After this phase, until the calves are weaned, reported mortality ranges between 5 and 11% [11].

Two-thirds of all calf diseases occur in the first 4 weeks of life [12]. The most common diseases are gastrointestinal infections, umbilical and joint infections, and pneumonia [8]. Studies on a cohort of heifer calves over the course of the first 9 weeks of life revealed disease frequencies of 48.2% diarrhea, 45.9% pneumonia, and 28.7% omphalitis [13].

While postnatal measures such as optimal colostrum supply [14], appropriate feeding [15], housing [16], and, if necessary, de-worming [17] are known to have an important influence on calf health, preventive management antepartum is not established on many farms. The aim of preventive management is to recognize disruptive factors of calf health and to avoid or reduce their negative effects. In recent years, studies have been conducted to record the effects of dam heat stress, body condition, vaccination, parity, and twin pregnancy on calf morbidity and mortality [18,19,20,21,22]. Individual studies often encompass a limited number of herds with similar management practices, climatic conditions, and genetic backgrounds. This might limit the inference of a treatment effect and shows the need for a systematic review. The effect of dam nutrition during gestation on colostrum quality, calf health, and perinatal mortality has been recently reviewed by Mee [23]. As this was created as a review of the narrative type, reporting contrary results, there seems also to be a need for a systematic review. The objectives of this paper were to perform a systematic review of the literature over the last 24 years to evaluate the influence of dam heat stress, nutrition, body condition, vaccination, parity, and twin pregnancy on calf morbidity and mortality.

## 2. Materials and Methods

A review protocol was created in accordance with Preferred Reporting Items for Systematic Reviews and Meta-Analyses (PRISMA)-P guidelines [24]. The search strategy was defined based on PICO (Population, Intervention, Comparator, Outcome) terms. Considering the passive transfer of immunity, the risk of calf morbidity, and the risk of calf mortality used as outcomes, the population is dairy calves, and the interventions were dam gestational heat stress, nutritional supplementations or feeding regimes, deviations from the recommended body condition score, vaccination, first parity, and twin pregnancies, while dams without heat stress, fed regularly, with recommended body condition score, without vaccination, multiparous dams, and dams with singletons acted as a comparator. Only peer-reviewed articles that presented primary research with either an experimental or observational study design were included in this review. In addition, only studies in English or German were included, and the full text had to be available online or through Justus-Liebig University library. Studies from outside the warm temperate zone (C) and the snow zone (D) in the Köppen–Geiger climate classification [25] were excluded to have a more similar basis, as climatic conditions have an influence on reproductive traits in dairy cattle [26]. This led mainly to the inclusion of countries in the European Union, North and Central America, and Australasia. Dairy breeds were defined as typically farmed Holstein-Friesian, Norwegian Red, Brown Swiss, Ayrshire, Simmental, or Jersey breeds [27].

In this review, mortality was divided into two phases according to Compton et al. [11]: from birth to 48 h of life (perinatal mortality) and from 48 h of life to weaning. Morbidity was assessed up to weaning.

Literature searches were conducted in Web of Science, CAB abstracts, and PubMed databases on 10 February 2024, and again on 20 December 2024, with restrictions to publication dates after 2000. This restriction was chosen because studies performed before 2000 do not represent the actual management and feeding practices [28,29]. Table 1 summarizes the search categories and search terms used for the antepartum factors. No difference was observed in the number of articles found, whether “calf” or “calves” and “dairy cows” or “dairy cattle” were used as search terms. Next, publications identified by this process were checked for additional references that had not been identified by the initial search process.

Studies were exported into Excel (Microsoft Corporation, Redmond, WA, USA). Duplicate results were documented and removed, and the remaining studies were subjected to two rounds of screening conducted by two authors.

In the first round of screening, titles, and abstracts were assessed for relevance using the following questions: (1) Does the title or abstract describe a study involving dairy cattle? (2) Does the title or abstract describe an experimental or observational study design? (3) Does the title or abstract include at least one of the topics in Table 1? Studies were excluded if one or more of these criteria were not fulfilled.

During the second round of screening, full-text scans were completed on the remaining studies using the following questions: (1) Does the study examine the influence of its subject matter on morbidity or mortality in dairy calves? The remaining studies had to be validated by three co-authors. An agreement of three out of five authors led to the inclusion of the paper.

Study-level data included publication year, country, study design, and study period (season). Population characteristics included sample size, breed, production type, length of experimental period, housing type, and detailed descriptions of treatments. Outcome measures, methodology, and conclusions were extracted from each paper with Excel using a standardized spreadsheet. To minimize errors in data extraction, two authors performed data extraction separately. The conclusions were based on reported statistics, with significance declared at *p* ≤ 0.05. When possible, the mean and standard error values of each treatment group were extracted. We present conclusions as described by the authors and the reported direction of the statistically significant effect, with “+” indicating a positive or desirable effect, “=” indicating no effect or neutral effect, and “–” indicating a negative or undesirable effect.

## 3. Results and Discussion

To determine factors that have a lasting influence on calf morbidity and mortality, the time of exposure (“time at risk”) must be defined [30]. One problem with the comparability of the studies is that many authors defined the time of exposure differently [11]. For example, the term “perinatal mortality” is used from 1 h [31], to 24 h [20], or up to 48 h [10] after birth, which possibly leads to different outcomes because other factors may play a role.

### 3.1. Heat Stress During Late Gestation

#### 3.1.1. Study Selection

**Figure 1:**
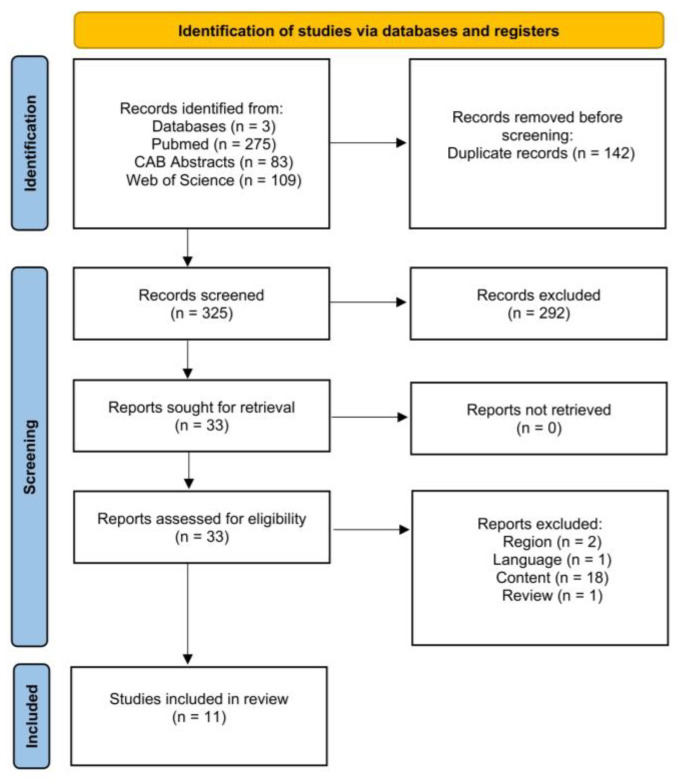
Flow diagram for the systematic review of studies investigating the effect of heat stress on calf morbidity and mortality, showing the number of studies that were screened, assessed for eligibility, and included in the systematic review according to [32].

Figure 1 is a PRISMA flow chart [32] showing the number of studies that were screened, assessed for eligibility, and included in the review, with reasons for exclusions at each stage. Of the 325 articles that were initially screened by title and abstract, 33 full texts were reviewed, and 22 studies did not fulfill the inclusion criteria. The remaining 11 articles were eligible as they investigated the effect of antepartum heat stress on our outcomes of interest. Therefore, data extraction was performed for 15 outcomes from 11 studies.

#### 3.1.2. Study Characteristics

A detailed overview of the characteristics of each study is provided in Table 2. The studies were performed in six countries: the USA (*n* = 6; 55%), China (*n* = 1; 9%), Germany (*n* = 1; 9%), Mexico (*n* = 1; 9%), Serbia (*n* = 1; 9%), and Türkiye (*n* = 1; 9%). The number of herds varied from 1 to 53, and the number of cows ranged from 20 to 21,316. The selection criteria for study enrolment on the cow and/or herd level were reported in all the papers. All studies were published after 2014.

One of the greatest challenges for future agriculture in the face of progressive climate change is increasing heat stress in many parts of the world [43,44]. The effects of heat stress on dairy cattle performance [45,46], well-being [46], and male and female reproductive performance [47], even over generations [48,49], have been and are the subject of current research. The temperature–humidity index (THI) has proven to be a useful measure [46]. Depending on the author, the THI threshold value, above which heat stress is considered to occur in dairy cows, is defined differently. Threshold values of 60 to 78 have been published [50].

One of the studies investigated mortality and causes of death of calves in relation to antepartum heat stress (THI > 78 in the last 46 days before calving) of the dam [22]. No significant differences were observed in perinatal mortality and mortality of bull calves in the first 120 days of life. Similarly, no significant difference was observed in the death of heifer calves before the onset of puberty. Only the number of heifer calves leaving before puberty due to sickness, malformation, and growth retardation was significantly higher in the group with antepartum heat stress. The larger number of heifers that left before puberty due to sickness, malformation, and growth retardation may be due to epigenetic changes in pregnancy influenced by heat stress, as described in goats [51].

**Figure 2:**
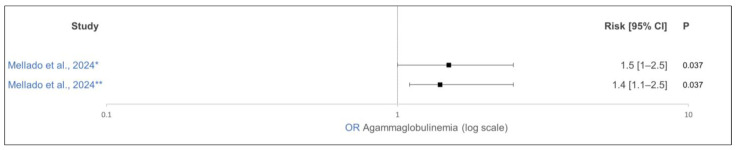
Effect of antepartum temperature–humidity index (THI) on nearly no transfer of IgG in calves. The odds ratio is shown as solid squares, and its 95% confidence interval is shown as whiskers. The control group’s THI was ≤70 in the last 90 days of gestation. * Average THI ≥ 80 in the last 90 days of gestation. ** Average THI of 70 to 80 in the last 90 days of gestation [36].

Several studies showed a significant negative effect of heat stress at the end of gestation on the transfer of immunoglobulins to the calves [33,34,35,36]. One study showed a significant weak negative correlation of −0.14 between the immunoglobulin transfer to the calves and the increase in THI by 1 in the last 60 days of gestation [35]. Mellado et al. [36] demonstrated a significantly increased odds ratio (OR) for the marginal transfer of immunoglobulins (IgG in the calves’ serum: approximately 0 g/L) to calves from cows that were exposed to heat stress 90 days before calving (Figure 2). In the calves that had received dam-sourced colostrum, the reduced colostrum quality due to heat stress in late gestation may have played a role here [33,52].

Studies that measured calf health using a calf health score found no significant difference between calves from cows with and without antepartum heat stress [37,38,39,40].

Tang et al. [41] found that calves from cows exposed to heat stress during the last 33 days of gestation had an increased incidence of diarrhea in the first 7 days of life. However, the control group was observed at a different time of the year; therefore, other factors may have influenced the frequency of diarrhea.

When analyzing the incidence of disease in calves and comparing the THI in the last 56 days of gestation, an increase in the incidence of 0.0277 (bronchopneumonia), 0.012 (diarrhea), and 0.0044 (omphalitis) per THI unit increase in the last week of gestation was observed [42]. Although significant, these increases were very low, and other factors have a much bigger influence on calf morbidity.

The increased incidence of disease in calves whose dams experienced heat stress in late gestation, as shown in two studies [41,42], could be due to altered macromolecule blood levels and reduced feed intake of the dams as a result of heat stress [53].

In summary, the duration and extent of heat stress showed a strong variation in the included studies. It seems that heat stress during pregnancy affects negatively the health of the calves at a low level in the short term. Avoidance of heat stress tends to have a positive effect on calf health. Further studies with comparable THI thresholds are needed, to gain more information on the impact on calf health.

### 3.2. Nutrition of the Pregnant Cow

#### 3.2.1. Study Selection

**Figure 3:**
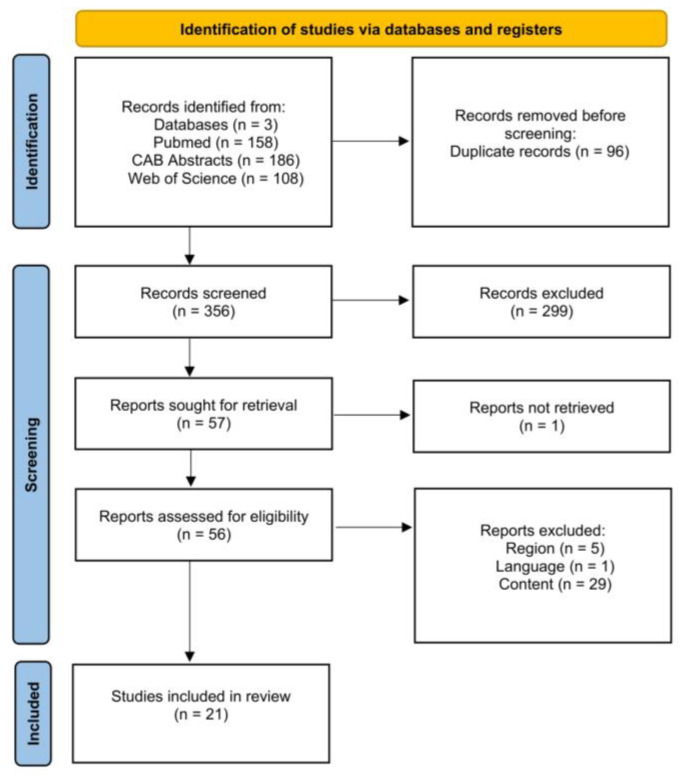
Flow diagram for the systematic review of studies investigating the effect of antepartum nutrition on calf morbidity and mortality, showing the number of studies that were screened, assessed for eligibility, and included in the systematic review according to [32].

Figure 3 is a PRISMA flow chart [32] showing the number of studies that were screened, assessed for eligibility, and included in the review, with reasons for exclusions at each stage. Of the 356 articles that were initially screened by title and abstract, 56 full texts were reviewed, with 35 studies not fulfilling the inclusion criteria. The remaining 21 articles were eligible as they investigated the effect of antepartum heat stress on our outcomes of interest. Therefore, data extraction was performed for 23 outcomes from 21 studies.

#### 3.2.2. Study Characteristics

A detailed overview of the characteristics of each study is provided in Table 3. The studies were performed in nine countries: the USA (*n* = 12; 52%), China (*n* = 3; 13%), Iran (*n* = 2; 9%), Hungary (*n* = 1; 4%), Germany (*n* = 1; 4%), Japan (*n* = 1; 4%), Belgium (*n* = 1; 4%), Cuba (*n* = 1; 4%), and Türkiye (*n* = 1; 4%). The number of cows ranged from 12 to 1511. The selection criteria for study enrolment on the cow and/or herd level were reported in all the papers. All studies were published after 2002.

The nutrition of dairy cows in mid-pregnancy is well known to play an important role in health and performance during pregnancy and in the subsequent lactation period, which is why various feeding concepts have been developed for this phase [75]. One part of the included studies focused on the effect of adding to the diet provitamins [54,55,56], rumen-protected essential amino acids [59,60,61], rumen-protected protein [62], betaine [63], choline [64], fat [65], essential fatty acids [66,67,68], zinc [57], chromium [58], magnesium butyrate [69], and selenium [70] on calf morbidity and mortality. The other part of the studies investigated the influence of diets negative in dietary cation–anion difference (DCAD) [71,72,73] and maternal energy supply [74] on calf health.

In late gestation, the effect of nutrition on calf mortality could not be determined [69,71,72]. Data on beef calves report mainly differences in calf birth weights and carcass characteristics due to maternal nutrition [76].

In human medicine, a lot of data exist on the effects of nutritional supplementation in pregnant women. Some of the studies have contradictory results, particularly due to discrepancies in whether the women had a deficient or sufficient provision of the supplemented nutrient [77,78]. Supplementation of methyl donors (e.g., betaine, choline, or methionine) during pregnancy showed a protective effect against metabolic diseases in human neonates [79].

Studies that investigated the influence of nutrition in late pregnancy on immunoglobulin transfer to calves showed a significant positive effect of adding the trace element selenium to the feed for 56 days before calving compared to a control group without additives [70]. This is in line with the finding of the improved transfer of passive immunity due to supplementation of selenium to colostrum [80] and underlines the importance of an adequate provision of selenium. The addition of rumen-protected betaine (in the last 28 days of gestation) [63], soybean oil, and fish oil (in the last 21 days of gestation) [65] also led to a significantly higher immunoglobulin transfer than in control groups without additives. In a study by Wang et al. [60], the addition of rumen-protected essential amino acids methionine and/or lysine in the last 21 days before calving resulted in improved immunoglobulin transfer to the calves. Feed additives showed no effect on the transfer of immunoglobulins in calves in other studies [54,55,56,57,58,62,64,66,67,68,69]. Although these studies are all designed as clinical trials and therefore show a high level of evidence, there are still wide variations in the duration and quantity of supplements used. Moreover, colostrum quality differs even in animals of one breed [81]. Based on the available data, no general recommendation on nutritional supplementation can be given to improve passive transfer of immunity.

Kovács et al. [69] found that calves from cows supplemented with magnesium butyrate for 23 days before calving had a significantly higher calf vitality score [82] at birth than that of the control group without supplementation. However, the incidence of disease up to weaning did not differ between the two groups.

**Figure 4:**
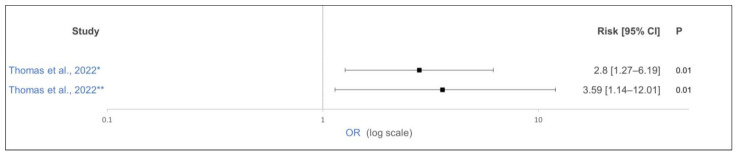
Effect of feeding rumen-protected lysine for 26 days antepartum on * medication (electrolytes and/or antibiotics) and ** antibiotic use in bull calves over the first 56 days of life. The odds ratio is shown as solid squares, and its 95% confidence interval is shown as whiskers. No supplementation of lysine was performed antepartum in the control group [61].

In bull calves up to 56 days of life, the control group showed an increased risk (OR: 2.8) of receiving medication (electrolytes and/or antibiotics) and an increased risk (OR: 3.69) of antibiotic use compared to that in the study group fed rumen-protected lysine 26 days before birth (Figure 4) [61]. In contrast, the addition of rumen-protected methionine for 28 days before calving had no effect on the fecal score in the first 63 days of the calves’ lives [59]. As interesting as the findings with the reduced medication and reduced antibiotics are, there is a need for more research to confirm the results on a larger scale.

In humans, an unbalanced maternal diet during pregnancy is associated with an increased risk of chronic diseases in affected children [83]. Therefore, DCAD feeding in the dry period, which is intended to reduce metabolic alkalosis or induce metabolic acidosis to reduce disease in dairy cattle in the transit phase [84], may result in negative effects on the calf. Two of the studies that investigated the effects of DCAD over 21 or 42 days before calving found no differences in immunoglobulin transfer, morbidity, and mortality of calves from experimental and control groups [71,72]. One study showed that heifer calves from cows fed DCAD 21 days before calving had an abnormal fecal score for significantly more days (7.93 days) in the first 70 days of life than heifer calves from cows in the control group (4.25 days) [73]. These results suggest no large influence of DCAD on calf morbidity and mortality.

In a study by García and González [74], calves from cows that consumed ≤75% of their energy requirements over the last 90 days of gestation were significantly more likely to have diarrhea up to the 90th day of life, compared to calves from cows that consumed ≥85% of their energy requirements during this period. In line with this, in human medicine, malnutrition during pregnancy also has negative effects on the health of children [77].

In summary, the diet of the pregnant cow is important for the development of a healthy calf after birth. However, we could not clarify from the available studies whether feeding supplements to the cow have any added benefits for the calves. Therefore, further investigations in larger animal groups are required to clarify this.

### 3.3. Body Condition of the Dam

#### 3.3.1. Study Selection

**Figure 5:**
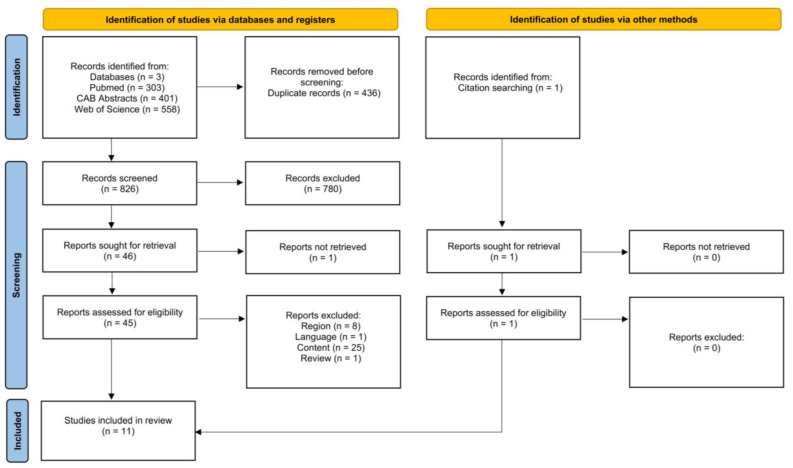
Flow diagram for the systematic review of studies investigating the effect of antepartum body condition score (BCS) on calf morbidity and mortality, showing the number of studies that were screened, assessed for eligibility, and included in the systematic review according to [32].

Figure 5 is a PRISMA flow chart [32] showing the number of studies that were screened, assessed for eligibility, and included in the review, with reasons for exclusions at each stage. Of the 826 articles that were initially screened by title and abstract, 46 full texts were reviewed, with 35 studies not fulfilling the inclusion criteria. The remaining 11 articles were eligible as they investigated the effect of antepartum body condition score (BCS) on our outcomes of interest. Therefore, data extraction was performed for 14 outcomes from 11 studies.

#### 3.3.2. Study Characteristics

A detailed overview of the characteristics of each study is shown in Table 4. The studies were performed in six countries: Germany (*n* = 4; 36%), Türkiye (*n* = 2; 18%), Hungary (*n* = 1; 9%), Iran (*n* = 1; 9%), Ireland (*n* = 1; 9%), New Zealand (*n* = 1; 9%), and the USA (*n* = 1; 9%). The number of herds varied from 1 to 567, and the number of cows ranged from 155 to 3445. The selection criteria for study enrolment on the cow and/or herd level were reported in all papers. All studies were published after 2007.

All included studies assessed the BCS on the day of calving. The categorization was made from 1 to 5 with 0.25 gradations between the individual scores [95]. The BCS at calving plays an important role in performance, health, fertility, and animal welfare during lactation [96]. Over-conditioned animals show an increased risk (OR: 1.27) of dystocia [87].

**Figure 6:**
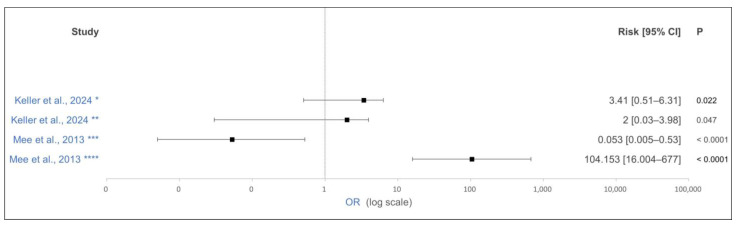
Effect of antepartum body condition score (BCS) on perinatal mortality of calves. The odds ratio is shown as solid squares, and its 95% confidence interval is shown as whiskers. * BCS < 3.25, control group BCS = 3.25–3.75; ** BCS > 3.75, control group BCS = 3.25–3.75; *** Heifer BCS ≥ 3.75, control group BCS ≤ 3; **** Heifer BCS = 3.25–3.5, control group BCS ≥ 3.75 [18,86].

Seven of the included studies reported on perinatal mortality of calves, with four studies covering a period of 48 h after calving [18,85,86,87] and three studies covering a period of 24 h after calving [88,89,90]. Mee et al. [86] and Keller et al. [18] demonstrated the effect of antepartum BCS on calf mortality (Figure 6). Mee et al. [86] demonstrated a protective effect of a BCS ≥ 3.75 compared to that of a BCS < 3, with an OR of 0.053 in dairy heifers. Heifers with a BCS of 3.25 to 3.75 had a significantly increased risk of perinatal mortality with an OR of 104.153. Keller et al. [18] showed, in contrast, that both over-conditioned (BCS > 3.75; OR: 2) and under-conditioned (BCS < 3.25; OR: 3.41) cows had an increased risk of perinatal mortality. The other studies were unable to demonstrate a significant effect of BCS on perinatal mortality [85,87,88,89,90].

One difficulty in comparing different studies is the subjective assessment of the BCS. Kristensen et al. [97] found little agreement in the assessment of BCS without training the examiners. The large variation in the 95% confidence interval for perinatal mortality in the study by [86] can be explained by the assessment of BCS by farmers and not by a standardized team of investigators; therefore, the results should be assessed with caution. In addition, only first-parity animals were included.

Furthermore, the influence of the BCS of the dam on the immunoglobulin G (IgG) content in the calves’ serum 36 h after calving [91] and the total protein content on the third day of life [92] was investigated. Both studies showed an effect of the maternal BCS on the IgG content in the first colostrum but no effect on the IgG content or total protein content in the calves’ serum. As the BCS provides information about the metabolic health of dairy cows [96], the improved colostrum quality is not surprising.

A study by Kara [93] recorded a daily calf health score of 0 to 5 in calves over 28 days [98]. In dams with a BCS of 3 to 3.75, significantly more calves had scores of 0 and 1 (no health problems) during the study period. The postulated epigenetic effects of the energy level of dairy cows at different times of pregnancy on the offspring could also play a role here [99].

Meier et al. [94] investigated whether the BCS of the dam had an influence on the prevalence of omphalitis in calves. They found a significant influence of over-conditioned (OR: 1.37) and under-conditioned (OR: 1.38) dams on the occurrence of omphalitis. Here, the BCS of the dams could be seen as an indicator of the general potential for improvement in farm management and to a lesser extent as a direct influence on calf health [18].

In conclusion, the BCS of the dam plays an important role in perinatal mortality in calves, and the results of this systematic review show that the recommended BCS antepartum of 3.25 to 3.75 seems to be adequate.

### 3.4. Vaccination of the Dam

#### 3.4.1. Study Selection

**Figure 7:**
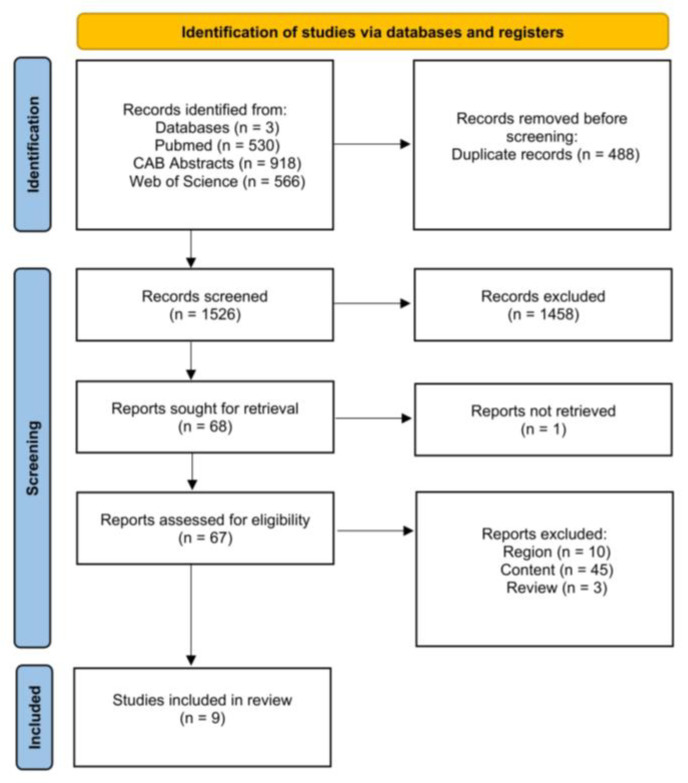
Flow diagram for the systematic review of studies investigating the effect of dam vaccination on calf morbidity and mortality, showing the number of studies that were screened, assessed for eligibility, and included in the systematic review according to [32].

Figure 7 is a PRISMA flow chart [32] showing the number of studies that were screened, assessed for eligibility, and included in the review, with reasons for exclusions at each stage. Of the 1526 articles that were initially screened by title and abstract, 68 full texts were reviewed, with 59 studies not fulfilling the inclusion criteria. The remaining nine articles were eligible, as they investigated the effect of vaccination of the dam on our outcomes of interest. Therefore, data extraction was performed for 15 outcomes from nine studies.

#### 3.4.2. Study Characteristics

A detailed overview of the characteristics of each study is provided in Table 5. The observations were performed in seven countries: the USA (*n* = 3; 33%), the Netherlands (*n* = 2; 22%), Belgium (*n* = 1; 11%), Canada (*n* = 1; 11%), Estonia (*n* = 1; 11%), Germany (*n* = 1; 11%), and New Zealand (*n* = 1; 11%). The number of herds varied from 5 to 13,000, and the number of cows ranged from 523 to 11,465. Two studies were on the herd level. The selection criteria for study enrolment on the cow and/or herd level were reported in all papers. All studies were published after 2007.

Vaccination of the dam against specific pathogens during pregnancy aims to ensure that increased levels of immunoglobulins against these pathogens circulate in the maternal blood and are transferred to the bovine neonate via colostrum, thereby inducing passive immunity [19]. To achieve adequate immunity, the calf must absorb an adequate quantity of antibodies via the colostrum.

In the included studies, the influence of vaccination of the dam against pathogens causing calf diarrhea [19,92,100,101,103,104] and those causing bovine respiratory disease (BRD) [102,105,106] on the morbidity and mortality of calves was investigated. Four of the studies investigated the influence of vaccination of dams on calf mortality. Viidu and Mõtus [19] found a reduction in calf mortality within the first 21 days of life when vaccinations against calf diarrhea pathogens were carried out correctly, compared to the previous year without vaccination. The risks differed between farms that fed their calves transition milk for more than 14 days (hazards ratio [HR]: 0.72) and those that fed transition milk for less than 14 days (HR: 0.24). The positive effect of vaccination may be buffered by the effect of longer transition milk feeding, which has a positive effect on health [107]. The risk of calf mortality in the first 21 days of life increased compared to that in the previous year (HR: 1.61) on farms that did not vaccinate dams consistently or vaccinated contrary to the manufacturer’s instructions. One reason for inconsistent vaccination management could be that the target was set too ambitiously or not by the person responsible for its implementation, which can lead to inadequate implementation of the necessary steps [108].

Santman-Berends et al. [100] showed that vaccinating dams against calf diarrhea pathogens (*E. coli*, rota, and corona virus) reduces the risk of postnatal calf mortality (3rd–14th day of life) (incidence rate ratio [IRR] of 0.91). This effect was no longer detectable in the mortality of calves from the 15th to the 55th day of life; in fact, the vaccination group had an increased risk of mortality (IRR: 1.07). This increased risk was quite low. The data collection in this study was performed on a large scale, without asking for the motivation and correct implementation of vaccination and for the presence of other underlying courses. Meganck et al. [101] found no difference in calf mortality in the first 21 days of life between the group of calves whose dams were vaccinated against calf diarrhea and who were treated with halofuginone lactate in the first 7 days and the untreated control group.

**Figure 8:**
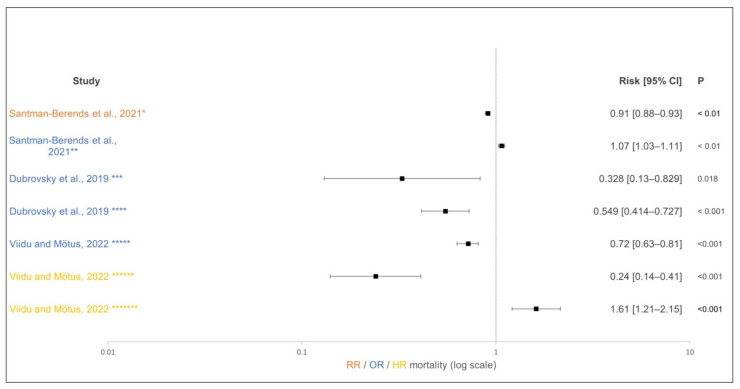
Effect of dam vaccination on calf mortality. The effect estimate is shown as solid squares, and its 95% confidence interval is shown as whiskers. * Vaccination of dams against calf diarrhea and postnatal mortality (3–14 days); ** vaccination of dams against calf diarrhea, preweaning mortality (15–55 days); *** vaccination of dams against bovine respiratory disease (BRD), mortality due to BRD; **** vaccination of dams against BRD, overall mortality; ***** vaccination of dams against calf diarrhea + ≥14 days feeding on transition milk, mortality due to diarrhea (21 days); ****** vaccination of dams against calf diarrhea + <14 days feeding on transition milk, mortality due to diarrhea (21 days); ******* no consistent vaccination of dams against calf diarrhea, mortality due to diarrhea (21 days) [19,100,102].

Vaccination of dams with a modified live BRD vaccine reduced both mortality associated with BRD in calves (OR: 0.328) and mortality up to weaning (OR: 0.549) compared with calves from unvaccinated dams [102] (Figure 8). The so-called non-specific effects of vaccination may play a possible role in mortality. For some vaccines, positive or negative effects have been described independently from the intended effect [109].

Positive effects of maternal vaccination on the non-vaccine-specific immunoglobulin M content in colostrum have been described [110]. The expectation might be that calves from vaccinated cows benefit from a higher transfer of passive immunity. However, one study showed that maternal vaccination against neonatal diarrhea had no effect on the immunoglobulin transfer to calves [92]. In this study, only a quantitative comparison of passive transfer between herds with and without vaccination was performed, and there was a lack of information on whether there was a change in passive transfer in the herds compared to the time before implementing the vaccination. Moreover, the presence of specific antibodies was not determined.

One study showed that vaccination of dams against calf diarrhea in combination with metaphylactic halofuginone lactate treatment for the first 7 days reduced the risk of calf diarrhea (OR: 0.26) [101]. The extent of the influence of dam vaccination and halofuginone lactate treatment cannot be determined individually. Halofuginone lactate reduces oocyst excretion of *Cryptosporidium parvum*, diarrhea incidence, and mortality in calves up to 28 days of age when applied before the onset of symptoms [111]. A similar conclusion was reached in a study that reported the occurrence of liquid feces in 9- to 21-day-old calves. The risk in calves from vaccinated dams was significantly lower (OR: 0.2) than that in calves from unvaccinated dams [104]. Another study found no effect of dam vaccination on the occurrence of calf diarrhea up to the 30th day of life [103].

Data on protection against BRD in calves by vaccinating dams with live or inactivated vaccines are also inconclusive. Dubrovsky et al. [106] demonstrated the protective effect of an inactivated vaccine (HR: 0.847) and a live vaccine (HR: 0.326) in calves up to weaning, compared with calves from unvaccinated cows. In contrast, Maier et al. [105] were unable to demonstrate any influence of the use of an inactivated or live vaccine in the dams on the incidence of disease in the calves up to weaning. Here, the consistency [19] and motivation [108] for vaccination were not recorded. In addition, BRD is dependent on various environmental factors [112,113].

In summary, vaccination of dams during pregnancy has a positive effect on the calves, although this is difficult to assess as interactions with many other factors exist, and good colostrum uptake must be ensured to achieve an effect.

### 3.5. Parity

#### 3.5.1. Study Selection

**Figure 9:**
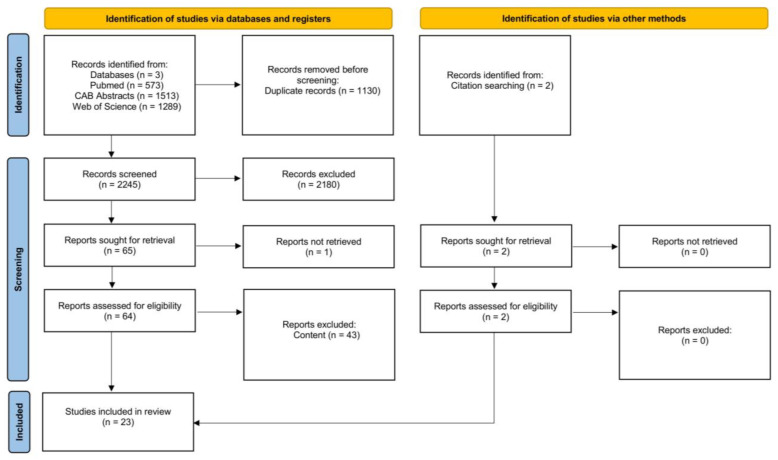
Flow diagram for the systematic review of studies investigating the effect of parity on morbidity and mortality, showing the number of studies that were screened, assessed for eligibility, and included in the systematic review according to [32].

Figure 9 is a PRISMA flow chart [32] showing the number of studies that were screened, assessed for eligibility, and included in the review, with reasons for exclusions at each stage. Of the 2245 articles that were initially screened by title and abstract, 64 full texts were reviewed, and 43 studies did not fulfill the inclusion criteria. Two articles were found via citation searching. The remaining 23 articles were eligible as they investigated the effect of parity on our outcomes of interest. Therefore, data extraction was performed for 53 outcomes from 23 studies.

#### 3.5.2. Study Characteristics

A detailed overview of the characteristics of each study is shown in Table 6. The studies were performed in seven countries: Iran (*n* = 6; 26%), the USA (*n* = 4; 17%), Germany (*n* = 3; 13%), England (*n* = 2; 9%), Ireland (*n* = 1; 4%), Japan (*n* = 1; 4%), Lithuania (*n* = 1; 4%), New Zealand (*n* = 1; 4%), Norway (*n* = 1; 4%), Sweden (*n* = 1; 4%), the Netherlands (*n* = 1; 4%), and Türkiye (*n* = 1; 4%). The number of herds varied from 1 to 14,423, and the number of cows ranged from 392 to 1,281,737. The selection criteria for study enrolment on the cow and/or herd level were reported in all the papers. All studies were published after 2000.

**Figure 10:**
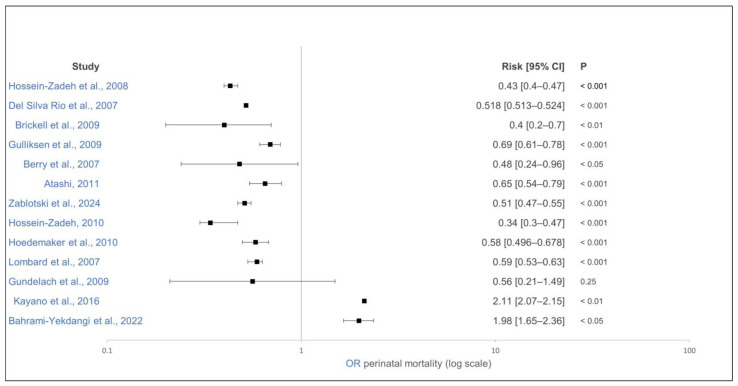
Effect of dam parity on perinatal mortality of calves. The effect estimate is shown as solid squares, and its 95% confidence interval is shown as whiskers. Parity ≥ 2, control group parity = 1 [10,20,21,85,87,88,114,115,117,118,119,122,124].

Most of the studies found a lower perinatal mortality rate in calves from multiparous cows than that of calves from primiparous cows, regardless of whether a 48 h after calving period or less was used (Figure 10) [21,114,124]. Two studies found no difference in calf mortality rates 1 h after calving [31] or 24 h after calving [88] between primiparous and multiparous cows. However, two studies found a higher calf mortality rate 48 h after calving in multiparous cows than that in primiparous cows [87,119]. When comparing cows with a parity of ≥3 to cows with a parity of 2, one study showed a lower calf mortality rate in the first 24 h after calving [120], while one study showed a higher calf mortality rate in the first 48 h after calving [10].

The higher perinatal mortality of calves from primiparous cows recorded in most studies has many possible causes that can be influenced by management, such as age at first calving, choice of bull, and birth monitoring. All these factors influence the dystocia rate [130] and perinatal mortality [131]. The dystocia rate is higher in primiparous cows than in multiparous cows [132]. Dystocia increases the risk of perinatal mortality by 3.37 for moderate dystocia and 17.7 for high-grade dystocia [10]. Birth monitoring of cows in first parity was not carried out on 68% of farms in one study [18]. The implementation of birth monitoring in primiparous cows showed a negative correlation with perinatal mortality [18].

The effect of parity on mortality decreases with an increase in the defined time at risk [115,118,125]. No difference was detected between the mortality of calves from primiparous or multiparous cows in the first 90 days of life [125]. The mortality of heifer calves from primiparous cows over a period of 120 days after calving was significantly increased compared to heifer calves from multiparous cows. If perinatal mortality (24 h after calving) was not considered, the effect was no longer significant [115]. In this study, the risk of mortality of heifer calves from primiparous cows in the first 30 days of life tended to be increased (*p* = 0.07). In another study, mortality in the period between 24 h after calving and 7 days after calving was increased in calves from primiparous cows, compared to that of calves from multiparous cows. This effect was also detectable between 8 days after calving and 30 days after calving [118].

One study found no effect of maternal parity on the immunoglobulin transfer of calves [126], although the colostrum quality of primiparous animals is often lower than that of multiparous animals [81].

In one study, the overall morbidity in the first 120 days of life was lower in heifer calves from primiparous cows than in calves from multiparous cows [115]. Similarly, in this study, the morbidity of respiratory diseases was lower in calves from primiparous cows than in calves from multiparous cows during the same period. The same conclusion was reached in another study that investigated the first 90 days of life [128]. A study that investigated respiratory diseases over 90 days in heifer calves was unable to determine any difference in frequency in relation to the parity of the dam [127].

Two studies found no difference in the incidence of diarrhea in relation to maternal parity over a period of 90 days of life [127,128]. One study showed a lower risk of diarrhea in heifer calves from primiparous cows than in calves from multiparous cows in the first 120 days of life [115]. Possible explanations for the lower disease incidence of calves from primiparous cows could be the lower metabolic stress of heifers during pregnancy with less negative impact on the unborn calf.

In a study, the incidence of omphalitis in the first 7 days of life was lower in calves from cows in their second parity than in calves from cows in their third parity [129].

In summary, parity plays an important role in perinatal mortality. Since parity cannot be influenced, other management factors play an important role, such as birth monitoring and early detection and treatment of dystocia. The findings of lesser morbidity of calves from primiparous cows in two studies is quite interesting and needs further research.

### 3.6. Twin Pregnancy

#### 3.6.1. Study Selection

**Figure 11:**
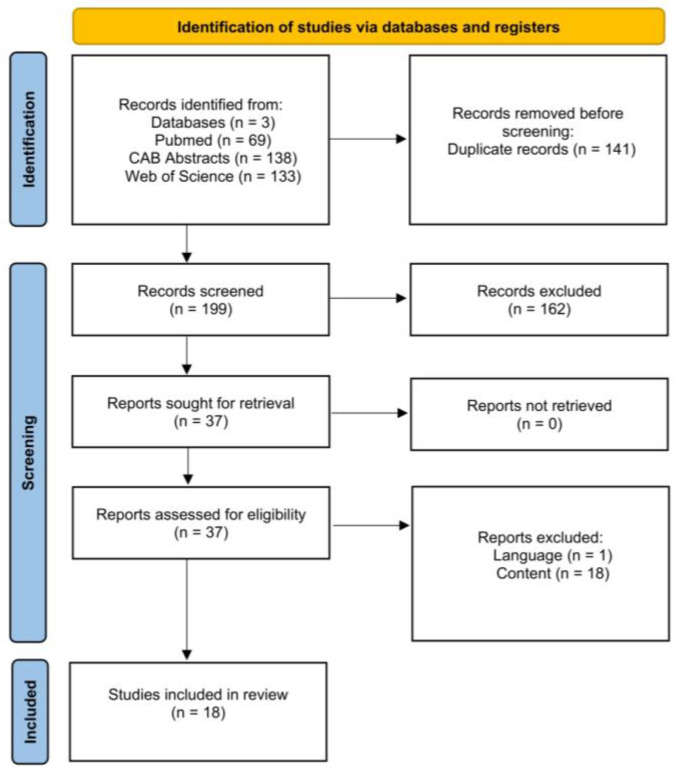
Flow diagram for the systematic review of studies investigating the effect of twin pregnancy on calf morbidity and mortality, showing the number of studies that were screened, assessed for eligibility, and included in the systematic review according to [32].

Figure 11 is a PRISMA flow chart [32] showing the number of studies that were screened, assessed for eligibility, and included in the review, with reasons for exclusions at each stage. Of the 199 articles that were initially screened by title and abstract, 37 full texts were reviewed, and 19 studies did not fulfill the inclusion criteria. The remaining 18 articles were eligible as they investigated the effect of twin pregnancy on our outcomes of interest. Therefore, data extraction was performed for 20 outcomes from 18 studies.

#### 3.6.2. Study Characteristics

A detailed overview of the characteristics of each study is given in Table 7. The observations were performed in ten countries: Iran (*n* = 4; 22%), the USA (*n* = 4; 22%), England (*n* = 2; 11%), Ireland (*n* = 2; 11%), the Czech Republic (*n* = 1; 6%), Germany (*n* = 1; 6%), Japan (*n* = 1; 6%), Mexico (*n* = 1; 6%), New Zealand (*n* = 1; 6%), and Norway (*n* = 1; 6%). The number of herds varied from 1 to 29,299, and the number of cows ranged from 392 to 11,256,112. The selection criteria for study enrolment on the cow and/or herd level were reported in all the papers. All the observations were published after 2004.

**Figure 12:**
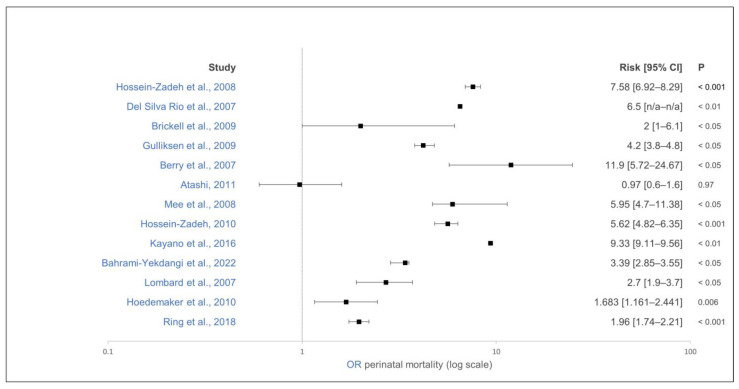
Effect of twin pregnancy on perinatal mortality of calves. The effect estimate is shown as solid squares, and its 95% confidence interval is shown as whiskers. OR of perinatal mortality, control group: singletons [20,21,85,87,114,115,116,117,118,119,122,124,134].

Twin pregnancies result in increased rates of pregnancy loss, shortened gestation periods, and increased dystocia rates [136]. Twin rates of up to 20% have been reported in dairy cows in the first month of pregnancy [137]. Almost all studies found a lower perinatal mortality rate in singleton calves than in twin calves, regardless of whether a 48 h after calving period or less was used (Figure 12) [20,21,85,87,114,115,116,117,118,119,124,133,134]. The calculated ORs were between 1.683 and 13.36. The highest OR was recorded in primiparous cows with twins [116]. Only one study could not demonstrate an effect of twin birth on perinatal mortality up to 48 h after calving [122]. Mortality in the first week of life [118] and up to weaning [102] was also significantly higher in twins than in singletons. This underlines the importance of detecting twin pregnancies during regular pregnancy checks [136] and correspondingly implementing more intensive birth monitoring of twin pregnant cows [92].

Two studies found no influence of twin births on the transfer of immunoglobulins [126] and the failure of passive transfer [135] in calves. One study showed a higher chance (OR: 1.4) of adequate transfer of immunoglobulins in single-born calves than in twin births [36]. The efforts of calf caretakers to care for weak calves certainly also play a role here [104].

In summary, twin pregnancies play an important role in perinatal mortality. The recognition of twin pregnancies and appropriate birth management are important. Data on diseases associated with twin births are not available in the present studies, which suggests that twins that survive the perinatal phase have no detectable differences present, compared with calves born singly.

### 3.7. Methodological Strengths and Limitations

Although a meta-analysis can provide quantitative synthesis, it was not feasible in the present review due to substantial heterogeneity among the included studies. Differences in study design, outcome definitions, time points for assessing morbidity and mortality, management systems, breeds, and environmental conditions created a high degree of methodological and clinical variability. Moreover, key statistical data necessary for effect size calculation, such as standard deviations or confidence intervals, were frequently missing or inconsistently reported. Conducting a meta-analysis under these circumstances would risk producing misleading or biased results. Therefore, in accordance with PRISMA guidelines, we chose to present a qualitative systematic review, critically summarizing and interpreting the available evidence. This approach allows for a more accurate reflection of the current knowledge base and highlights important gaps for future research. The included studies were randomized clinical trials that, along with systematic reviews, provide the highest level of evidence and observational studies, which provide a much lower level of evidence, but can also be used for evaluating on-farm interventions [138]. This selection led to a lower level of evidence, but due to the restricted number of randomized clinical trials in this field, this limitation must be tolerated. Another reason why this review may not reflect the entire peer-reviewed literature is linguistic limitation, as it occurs often in reviews [139]. However, considering the low exclusion number regarding language, the probability of missing an important article is quite low. Although it is possible to miss relevant earlier studies, because this review focused on studies conducted over the last 24 years, in our opinion, this reflects recent genetics and farm management practices that are relevant for modern dairy farming and can, therefore, provide an increased grade of evidence for current conditions. Another limitation of this systematic review is the exclusion of studies performed outside the warm temperate zone (C) and the snow zone (D) in the Köppen–Geiger climate classification. As it is known that different climatic zones have an influence on reproductive traits in cattle [26], we aimed for comparable climatic conditions to have comparable studies. There might be some more insights by adding tropical or hot arid conditions, but the results would have to be used with caution.

## 4. Conclusions

Evaluation of the literature shows that all antepartum factors investigated have a relevant influence on calf morbidity and mortality. As intrapartum and postpartum factors also have effects on calf morbidity and mortality, most effects of antepartum factors cannot be interpreted solely. Discrepancies exist as to whether these are predetermined by the animal (parity and twin pregnancy), environmental factors (heat stress), or management (nutrition, body condition, and vaccination of the dam). Nevertheless, ways to reduce calf morbidity and mortality for all the factors investigated can be identified. The most important measures are the identification of risk factors and appropriate countermeasures. While animal factors such as parity and twin pregnancy can be easily assessed and countermeasures such as improved birth monitoring are theoretically easy to implement, they often fail because of the additional work. Further research can possibly show if Artificial Intelligence can partly solve this problem of contradiction between knowledge and realization. Regarding heat stress, this review cannot give advice except for the vague call for a reduction in heat stress during pregnancy. More studies with a common definition of a threshold for heat stress are needed. The analysis of the management factors (nutrition, body condition, and vaccination of the dam) shows that good farming practice seems to be adequate for the reduction in morbidity and mortality of dairy calves.

## Figures and Tables

**Table 1:** Search categories and terms for the antepartum risk factors associated with morbidity and mortality in dairy calves.

Search Categories	Search Term
Heat stress of the dam	dairy AND cow AND heat stress OR climate OR summer AND gestation AND calf
Nutrition of the dam	dairy AND cow AND nutrition AND gestation AND calf
Body condition of the dam	dairy AND cow AND bcs OR body condition AND calf
Vaccination of the dam	dairy AND cow AND vaccination OR immunity AND calf
Parity	dairy AND cow AND parity OR lactation AND calf
Twin pregnancy	dairy AND cow AND twin pregnancy AND calf OR twins OR twinning AND calf

**Table 2:** Studies investigating the effect of heat stress on calf morbidity and mortality. The reported direction of the statistically significant effect, with “=” as no effect or neutral effect, and “−” as a negative or undesirable effect.

Author	Country	Study Design	Animals/ Herds	Breed	Outcome	Study Group	Control Group	Effect Estimate (95% CI)	*p*	Result	Comment
[22]	USA	clinical trial	146	Holstein	Perinatal mortality (48 h after calving)	THI > 78 last 46 d of gestation	cows cooled last 46 d of gestation	−	0.25	=	−
[22]	USA	clinical trial	146	Holstein	Mortality bull calves up to 4 months	THI > 78 last 46 d of gestation	cows cooled last 46 d of gestation	−	0.35	=	−
[22]	USA	clinical trial	146	Holstein	Heifers leaving before puberty	THI > 78 last 46 d of gestation	cows cooled last 46 d of gestation	−	0.26	=	−
[22]	USA	clinical trial	146	Holstein	Heifers being culled before puberty due to sickness, malformation, growth retardation	THI > 78 last 46 d of gestation	cows cooled last 46 d of gestation	−	0.03	**-**	−
[33]	Serbia	Observation	20/1	Holstein	IgG calf serum (24 h after birth)	THI ~ 75 last 53 d of gestation	THI ~ 44 last 53 d of gestation	−	0.036	**-**	−
[34]	USA	Clinical trial	19/1	Holstein	IgG calf serum (56 d after birth)	THI ~ 77.3 last 60 d of gestation	cows cooled last 60 d of gestation	−	0.03	**-**	only heifer calves
[35]	Türkiye	Observation	878/1	Holstein	Correlation with serum brix (32–48 h after calving)	THI last 60 d of gestation	−	r = −0.14	<0.001		higher THI results in lesser serum brix
[36]	Mexico	Observation	4411/1	Holstein	Agammaglobulinemia	average THI ≥ 80 last 90 d of gestation	average THI ≤ 70 last 90 d of gestation	OR 1.5 (1.0–2.5)	0.037	**-**	−
[36]	Mexico	Observation	4411/1	Holstein	Agammaglobulinemia	average THI 70–80 last 90 d of gestation	average THI ≤ 70 last 90 d of gestation	OR 1.4 (1.1–1.8)	0.037	**-**	−
[37]	USA	Clinical trial	36	Holstein	Calf Health Score	THI > 78 last 46 d of gestation	cows cooled last 46 d of gestation	−	−	=	−
[38]	USA	Clinical trial	60	Holstein	Calf Health Score	THI > 72 last 46 d of gestation	cows cooled last 46 d of gestation	−	−	=	−
[39]	USA	Clinical trial	36	Holstein	Calf Health Score	THI > 78 last 46 d of gestation	cows cooled last 46 d of gestation	−	−	=	−
[40]	USA	Clinical trial	36	Holstein	Calf Health Score	THI > 78 last 44 ± 5 d of gestation	cows cooled last 44 ± 5 d of gestation	−	−	=	−
[41]	China	Observation	51/1	Holstein	Incidence of diarrhea first 7 days of life	THI 70–74 last 33 d of gestation	THI 57–67 last 33 d of gestation	−	−	−	different seasons
[42]	Germany	Observation	21,316/53	Holstein	Morbidity (Omphalitis (O), Diarrhea (D), Respiratory disease (RD))	THI last 56 d of gestation	−	−	−		last week of gestation increase of incidence 0.0044 (O), 0.012 (D), 0.0277 (RD) per increase of 1 THI unit

THI: Temperature-Humidity-Index; OR = Odds ratio.

**Table 3:** Studies investigating the effect nutrition of the pregnant cow on calf morbidity and mortality. The reported direction of the statistically significant effect, with “+” indicating the effect was interpreted as positive or desirable, “=” as no effect or neutral effect, and “−” as a negative or undesirable effect.

Author	Country	Study Design	Animals/Herds	Breed	Outcome	Study Group	Control Group	Effect Estimate (95% CI)	*p*	Result	Comment
[54]	USA	Clinical trial	18	Holstein	IgG calf serum (24 h after birth)	β-carotene supplementation in last 28 d of gestation	no supplementation	−	0.59	=	−
[55]	USA	Clinical trial	94	Holstein	Total protein calf serum (24 h after birth)	β-carotene supplementation in last 28 d of gestation	no supplementation	−	0.63	=	−
[56]	USA	Clinical trial	36	Holstein	IgG calf serum (24 h after birth)	Nicotinic acid supplementation in last 28 d of gestation	no supplementation	−	0.86	=	−
[57]	China	Clinical trial	40	Holstein	IgG calf serum (24 h after birth)	Zn-Methionine supplementation in last 60 d of gestation	no supplementation	−	−	=	−
[58]	Türkiye	Clinical trial	45	Holstein	IgG calf serum (24 h after birth)	Cr-Methionine supplementation in last 60 d of gestation	no supplementation or injection of levamisole	−	−	=	−
[59]	USA	Clinical trial	81	Holstein	Fecal score of the calves over first 9 weeks	Methionine supplementation in last 28 d of gestation	no supplementation	−	−	=	−
[60]	China	Clinical trial	120	Holstein	IgG calf serum (24 h after birth)	Methionine and/or Lysine supplementation in last 21 d of gestation	no supplementation	−	<0.01	+	Only heifer calves
[61]	USA	Clinical trial	78	Holstein	Medication in first 8 weeks of life (Electrolytes or antibiotics)	no supplementation	Lysine supplementation in last 26 d of gestation	OR 2.8 (1.27–6.19)	0.01	−	Only bull calves
[61]	USA	Clinical trial	78	Holstein	Antibiotics first 8 weeks of life	no supplementation	Lysine supplementation in last 26 d of gestation	OR 3.69 (1.14–12.01)	0.01	−	Only bull calves
[62]	Belgium	Clinical trial	74	Holstein	IgG calf serum (72 h after birth)	Rumen-protected protein supplementation in last 45 d of gestation	no supplementation	−	−	=	−
[63]	China	Clinical trial	24	Holstein	IgG calf serum (24 h after birth)	Rumen-protected betaine supplementation in last 45 d of gestation	no supplementation	−	<0.05	+	Only heifer calves
[64]	USA	Clinical trial	111	Holstein	IgG calf serum (24–36 h after birth)	Choline supplementation in last 28 d of gestation	no supplementation	−	−	=	−
[65]	Iran	Clinical trial	120	Holstein	IgG calf serum (24 h after birth)	Soybean oil or fish oil supplementation in last 21 d of gestation	no supplementation	−	<0.01	+	−
[66]	USA	Clinical trial	78	Holstein	IgG calf serum (24 h after birth)	Essential or conjugated fatty acids supplementation in last 56 d of gestation	no supplementation	−	0.09	=	−
[67]	USA	Clinical trial	96	Holstein	IgG calf serum (24 h after birth)	Essential fatty acids supplementation in last 56 d of gestation	no supplementation	−	0.31	=	−
[68]	Germany	Clinical trial	21	Holstein	Total protein calf serum (24 h after birth)	Conjugated linoleic acids supplementation in last 21 d of gestation	Fat supplementation	−	−	=	−
[69]	Hungary	Clinical trial	219	Holstein	Calf vitality at birth	Magnesium butyrate supplementation in last 23 d of gestation	no supplementation	−	0.001	+	−
[69]	Hungary	Clinical trial	219	Holstein	IgG calf serum; perinatal Mortality; Morbidity	Magnesium butyrate supplementation in last 23 d of gestation	no supplementation	−	−	=	−
[70]	USA	Clinical trial	60	Holstein	IgG calf serum (48 h after birth)	Selenium yeast supplementation in last 56 d of gestation	no supplementation	−	0.03	+	−
[71]	USA	Clinical trial	132	Holstein	IgG calf serum; Morbidity; Mortality;	Feeding DCAD- in last 22 of gestation	no DCAD	−	−	=	−
[72]	USA	Clinical trial	60	Holstein	IgG calf serum; Morbidity; Mortality;	Feeding DCAD- in last 21 or 42 d of gestation	no DCAD	−	−	=	−
[73]	Iran	Clinical trial	12	Holstein	Days with abnormal fecal score	Feeding DCAD- in last 21 d of gestation	no DCAD	−	<0.01	−	Only heifer calves
[74]	Cuba	Clinical trial	260	Holstein	Diarrhea (first 90 d of life)	≤75% of energy requirement in last 90 d of gestation	≥85% of energy requirement in last 90 d of gestation	−	<0.05	−	−

DCAD = diets negative in dietary cation-anion difference, OR = Odds ratio.

**Table 4:** Studies investigating the effect of BCS of the dam on calf morbidity and mortality. The reported direction of the statistically significant effect, with “+” indicating the effect was interpreted as positive or desirable, “=” as no effect or neutral effect, and “−” as a negative or undesirable effect.

Author	Country	Study Design	Animals/Herds	Breed	Outcome	Study Group	Control Group	Effect Estimate (95% CI)	*p*	Result	Comment
[85]	New Zealand	Observation	2384.1	Dairy	Perinatal mortality (48 h after calving)	BCS	−	−	−	=	−
[86]	Ireland	Observation	−0.30	Dairy	Perinatal mortality (48 h after calving)	BCS in Heifers ≥ 3.75	BCS in Heifers ≤ 3	OR 0.053 (0.005–0.53)	<0.0001	+	−
[86]	Ireland	Observation	−0.30	Dairy	Perinatal mortality (48 h after calving)	BCS in Heifers 3.25–3.5	BCS in Heifers ≥ 3.75	OR 104.153 (16.004–677)	<0.0001	−	−
[87]	Iran	Observation	14,546.3	Dairy	Perinatal mortality (48 h after calving)	BCS	−	−	−	=	−
[18]	Germany	Observation	−0.97	Dairy	Perinatal mortality (48 h after calving)	BCS < 3.25	BCS 3.25–3.75	OR 3.41 (0.51–6.31)	0.022	−	−
[18]	Germany	Observation	−0.97	Dairy	Perinatal mortality (48 h after calving)	BCS > 3.75	BCS 3.25–3.75	OR 2 (0.03–3.98)	0.047	−	−
[88]	Germany	Observation	411.1	Holstein	Perinatal mortality (24 h after calving)	BCS	−	−	−	=	−
[89]	Hungary	Observation	155.3	Holstein	Perinatal mortality (24 h after calving)	BCS	−	−	−	=	−
[90]	USA	Observation	1044.3	Holstein	Perinatal mortality (24 h after calving)	BCS	−	−	−	=	−
[91]	Türkiye	Observation	354.2	Holstein	IgG calf serum (36 h after birth)	BCS	−	−	−	=	−
[92]	Germany	Observation	551.124	Dairy	Total protein calf serum (3 d after birth)	BCS	−	−	−	=	−
[93]	Türkiye	Observation	517.1	Holstein	Calf Health Score 0 and 1 (good) until 28 d after calving.	BCS 3–3.75	BCS < 3 or BCS > 3.75	OR 1.59	0.001	+	−
[94]	Germany	Observation	3445.567	Dairy	Omphalitis	BCS < 3.25	BCS 3.25–3.75	OR 1.38 (1.06–1.79)	0.016	−	−
[94]	Germany	Observation	3445.567	Dairy	Omphalitis	BCS > 3.75	BCS 3.25–3.75	OR 1.37 (1.00–1.86)	0.045	−	−

OR = Odds ratio.

**Table 5:** Studies investigating the effect of vaccination of the dam on calf morbidity and mortality. The reported direction of the statistically significant effect, with “+” indicating the effect was interpreted as positive or desirable, “=” as no effect or neutral effect, and “−” as a negative or undesirable effect.

Author	Country	Study Design	Animals/Herds	Breed	Outcome	Study Group	Control Group	Effect Estimate (95% CI)	*p*	Result	Comment
[19]	Estonia	Observation	−/15	Dairy	Calf mortality due to diarrhea (21 d)	Vaccination of the dam against neonatal calf diarrhoe (NCD) + feeding transition milk at least 14 d	Same herds before implementing vaccination	HR 0.72 (0.63–0.81)	<0.001	+	−
[19]	Estonia	Observation	−/15	Dairy	Calf mortality due to diarrhea (21 d)	Vaccination of the dam against NCD + feeding transition milk up to 14 d	Same herds before implementing vaccination	HR 0.24 (0.14–0.41)	<0.001	+	−
[19]	Estonia	Observation	−/15	Dairy	Calf mortality due to diarrhea (21 d)	Vaccination of the dam against NCD not consequent	Same herds before implementing vaccination	HR 1.61 (1.21–2.15)	<0.001	−	−
[100]	Netherlands	observation	−/13,000	Dairy	Postnatal mortality (3–14 d)	Vaccination of the dam against NCD	No vaccination	IRR 0.91 (0.88–0.93)	<0.001	+	−
[100]	Netherlands	observation	−/13,000	Dairy	Preweaned mortality (15–55 d)	Vaccination of the dam against NCD	No vaccination	IRR 1.07 (1.03–1.11)	<0.01	−	−
[101]	Belgium, Netherlands	observation	523/24	Dairy	Mortality (first 21 d of life)	Vaccination of the dam against NCD + use of halofuginone lactate for 7 days	No vaccination, no halofuginone lactate	−	−	=	−
[102]	USA	observation	11,465/5	Dairy	Mortality due to BRD till weaning	Vaccination of the dam with a live vaccine against BRD	No vaccination	OR 0.328 (0.13–0.829)	0.018	+	−
[102]	USA	observation	11,465/5	Dairy	Overall mortality till weaning	Vaccination of the dam with a live vaccine against BRD	No vaccination	OR 0.549 (0.414–0.727)	<0.001	+	−
[92]	Germany	observation	551/124	Dairy	Total protein calf serum (3 d after birth)	Vaccination of the dam against NCD	No vaccination	−	−	=	−
[101]	Belgium, Netherlands	observation	523/24	Dairy	Morbidity NCD first 21 d of life	Vaccination of the dam against NCD + use of halofuginone lactate for 7 days	No vaccination, no halofuginone lactate	OR 0.26 (0.12–0.6)	<0.01	+	−
[103]	Canada	observation	1045/11	Dairy	Morbidity NCD first 30 d of life	Vaccination of the dam against NCD	No vaccination	−	−	=	−
[104]	New Zealand	observation	1283/97	Dairy	liquid faeces in 9-to-21-day-old calves	Vaccination of the dam against NCD	No vaccination	OR 0.2 (0.1–0.9)	0.03	+	−
[105]	USA	observation	4253/95	Dairy	BRD till weaning	Vaccination of the dam against BRD (live or inactivated)	No vaccination	−	−	=	−
[106]	USA	observation	11,300/5	Dairy	BRD till weaning	Vaccination of the dam with a modified live vaccine against BRD	No vaccination	HR 0.326 (0.263–0.405)	<0.001	+	−
[106]	USA	observation	11,300/5	Dairy	BRD till weaning	Vaccination of the dam with an inactivated vaccine against BRD	No vaccination	HR 0.847 (0.739–0.971)	<0.001	+	−

BRD = Bovine respiratory disease; HR = Hazard ratio; IRR = Incidence rate ratio; NCD = Neonatal calf diarrhea; OR = Odds ratio.

**Table 6:** Studies investigating the effect of parity on calf morbidity and mortality. The reported direction of the statistically significant effect, with “+” indicating the effect was interpreted as positive or desirable, “=” as no effect or neutral effect, and “−” as a negative or undesirable effect.

Author	Country	Study Design	Animals/Herds	Breed	Outcome	Study Group	Control Group	Effect Estimate (95% CI)	*p*	Result	Comment
[114]	Iran	Observation	104,572/16	Holstein	Perinatal mortality *	Parity = 2	Parity = 1	OR 0.43 (0.4–0.47)	<0.001	+	−
[114]	Iran	Observation	104,572/16	Holstein	Perinatal mortality *	Parity = 3	Parity = 1	OR 0.41 (0.37–0.45)	<0.001	+	−
[114]	Iran	Observation	104,572/16	Holstein	Perinatal mortality *	Parity ≥ 4	Parity = 1	OR 0.44 (0.41–0.48)	<0.001	+	−
[31]	Iran	Observation	2831/64	Dairy	Perinatal mortality (1 h after birth)	Parity ≥ 2	Parity = 1	−	−	=	−
[21]	USA	Observation	1,164,233/4103	Holstein	Perinatal mortality (24 h after birth)	Parity = 2	Parity = 1	OR 0.518 (0.513–0.524)	<0.001	+	−
[21]	USA	Observation	1,164,233/4103	Holstein	Perinatal mortality (24 h after birth)	Parity ≥ 3	Parity = 1	OR 0.526 (0.521–0.53)	<0.001	+	−
[115]	USA	Observation	7788/3	Holstein	Perinatal mortality (24 h after birth)	Parity ≥ 2	Parity = 1	OR 0.59 (0.53–0.63)	<0.001	+	−
[116]	Ireland	Observation	305,531/−	Holstein	Perinatal mortality (24 h after birth)	Parity ≥ 2	Parity = 1	−	<0.05	+	−
[117]	England	Observation	1097/19	Holstein	Perinatal mortality (24 h after birth)	Parity ≥ 2	Parity = 1	OR 0.4 (0.2–0.7)	<0.01	+	−
[118]	Norway	Observation	246,156/14,423	Dairy	Perinatal mortality (24 h after birth)	Parity = 2	Parity = 1	OR 0.69 (0.61–0.78)	<0.001	+	−
[118]	Norway	Observation	246,156/14,423	Dairy	Perinatal mortality (24 h after birth)	Parity = 3	Parity = 1	OR 0.7 (0.61–0.8)	<0.001	+	−
[118]	Norway	Observation	246,156/14,423	Dairy	Perinatal mortality (24 h after birth)	Parity ≥ 4	Parity = 1	OR 0.68 (0.59–0.78)	<0.001	+	−
[88]	Germany	Observation	463/1	Holstein	Perinatal mortality (24 h after birth)	Parity ≥ 2	Parity = 1	OR 0.56 (0.21–1.49)	0.25	=	−
[20]	Germany	Observation	13,158/46	Dairy	Perinatal mortality (24 h after birth)	Parity ≥ 2	Parity = 1	OR 0.58 (0.496–0.678)	<0.001	+	−
[119]	Japan	Observation	1,281,737/5,172	Dairy	Perinatal mortality (24 h after birth)	Parity ≥ 2	Parity = 1	OR 2.11 (2.07–2.15)	<0.01	−	−
[120]	Lithuania	Observation	3861/1	Holstein	Perinatal mortality (24 h after birth)	Parity ≥ 3	Parity = 2	OR 0.71 (0.47–0.972)	0.043	+	−
[121]	USA	Observation	666,341/−	Holstein	Perinatal mortality (48 h after birth)	Parity = 2 or 3	Parity = 1	−	<0.001	+	−
[85]	New Zealand	Observation	2384/1	Dairy	Perinatal mortality (48 h after birth)	Parity = 2	Parity = 1	OR 0.48 (0.24–0.96)	<0.05	+	−
[85]	New Zealand	Observation	2384/1	Dairy	Perinatal mortality (48 h after birth)	Parity = 3	Parity = 1	OR 0.43 (0.21–0.89)	<0.05	+	−
[85]	New Zealand	Observation	2384/1	Dairy	Perinatal mortality (48 h after birth)	Parity = 4	Parity = 1	OR 0.44 (0.26–0.75)	<0.05	+	−
[85]	New Zealand	Observation	2384/1	Dairy	Perinatal mortality (48 h after birth)	Parity ≥ 5	Parity = 1	OR 0.56 (0.35–0.89)	<0.05	+	−
[122]	Iran	Observation	12,283/1	Holstein	Perinatal mortality (48 h after birth)	Parity = 2	Parity = 1	OR 0.65 (0.54–0.79)	<0.001	+	−
[122]	Iran	Observation	12,283/1	Holstein	Perinatal mortality (48 h after birth)	Parity = 3	Parity = 1	OR 0.57 (0.45–0.74)	<0.001	+	−
[122]	Iran	Observation	12,283/1	Holstein	Perinatal mortality (48 h after birth)	Parity ≥ 4	Parity = 1	OR 0.75 (0.56–0.98)	0.04	+	−
[123]	Türkiye	Observation	947/1	Holstein	Perinatal mortality (48 h after birth)	Parity ≥ 2	Parity = 1	−	<0.05	+	−
[87]	Iran	Observation	51,405/3	Holstein	Perinatal mortality (48 h after birth)	Parity = 2	Parity = 1	OR 1.98 (1.65–2.36)	<0.05	−	−
[87]	Iran	Observation	51,405/3	Holstein	Perinatal mortality (48 h after birth)	Parity = 3	Parity = 1	OR 1.84 (1.48–2.29)	<0.05	−	−
[87]	Iran	Observation	51,405/3	Holstein	Perinatal mortality (48 h after birth)	Parity ≥ 4	Parity = 1	OR 2.2 (1.69–2.86)	<0.05	−	−
[10]	Germany	Observation	133,942/721	Dairy	Perinatal mortality (48 h after birth)	Parity = 2	Parity = 1	OR 0.51 (0.47–0.55)	<0.001	+	−
[10]	Germany	Observation	133,942/721	Dairy	Perinatal mortality (48 h after birth)	Parity ≥ 3	Parity = 1	OR 0.6 (0.56–0.64)	<0.001	+	−
[10]	Germany	Observation	133,942/721	Dairy	Perinatal mortality (48 h after birth)	Parity ≥ 3	Parity = 2	OR 1.18 (1.09–1.28)	<0.001	−	−
[124]	Iran	Observation	1,163,594/2552	Holstein	Perinatal mortality *	Parity = 2	Parity = 1	OR 0.34 (0.3–0.47)	<0.001	+	−
[124]	Iran	Observation	1,163,594/2552	Holstein	Perinatal mortality *	Parity = 3	Parity = 1	OR 0.31 (0.27–0.35)	<0.001	+	−
[124]	Iran	Observation	1,163,594/2552	Holstein	Perinatal mortality *	Parity ≥ 4	Parity = 1	OR 0.39 (0.36–0.43)	<0.001	+	−
[115]	USA	Observation	7788/3	Holstein	Mortality (1d–120 d)	Parity = 1	Parity ≥ 2	OR 0.9 (0.8–1.1)	0.375	=	only heifer calves
[115]	USA	Observation	7788/3	Holstein	Mortality (0 h–120 d)	Parity = 1	Parity ≥ 2	OR 1.2 (1.1–1.2)	<0.001	−	only heifer calves
[115]	USA	Observation	7788/3	Holstein	Mortality (0 h–30 d)	Parity = 1	Parity ≥ 2	HR 1.2 (1–1.4)	0.07	=	only heifer calves
[125]	Iran	Observation	4097/10	Holstein	Mortality (90 d)	Parity = 1	Parity ≥ 2	−	−	=	−
[118]	Norway	Observation	246,156/14,423	Dairy	Mortality (1d–7 d)	Parity = 2	Parity = 1	OR 0.9 (0.85–0.95)	<0.001	+	−
[118]	Norway	Observation	246,156/14,423	Dairy	Mortality (1d–7 d)	Parity = 3	Parity = 1	OR 0.89 (0.81–0.93)	<0.001	+	−
[118]	Norway	Observation	246,156/14,423	Dairy	Mortality (1d–7 d)	Parity ≥ 4	Parity = 1	OR 0.83 (0.78–0.89)	<0.001	+	−
[118]	Norway	Observation	246,156/14,423	Dairy	Mortality (8d–30 d)	Parity = 2	Parity = 1	OR 0.92 (0.87–0.98)	<0.05	+	−
[118]	Norway	Observation	246,156/14,423	Dairy	Mortality (8d–30 d)	Parity = 3	Parity = 1	OR 0.9 (0.84–0.97)	<0.05	+	−
[118]	Norway	Observation	246,156/14,423	Dairy	Mortality (8d–30 d)	Parity ≥ 4	Parity = 1	OR 0.85 (0.74–0.96)	<0.001	+	−
[126]	England	Observation	392/7	Holstein	IgG calf plasma (1–7 d after birth)	Parity	−	−	−	=	−
[115]	USA	Observation	7788/3	Holstein	Morbidity (120 d)	Parity = 1	Parity ≥ 2	OR 0.8 (0.7–0.9)	<0.001	+	Only heifer calves
[115]	USA	Observation	7788/3	Holstein	Respiratory disease (120 d)	Parity = 1	Parity ≥ 2	OR 0.8 (0.8–0.8)	<0.001	+	Only heifer calves
[127]	Sweden	Observation	3081/122	Dairy	Respiratory disease (90 d)	Parity	−	−	−	=	Only heifer calves
[128]	USA	Observation	449/3	Holstein	Respiratory disease (90 d)	Parity = 1	Parity ≥ 2	OR 0.29 (0.09–0.91)	0.03	+	−
[115]	USA	Observation	7788/3	Holstein	Diarrhea (120 d)	Parity = 1	Parity ≥ 2	OR 0.8 (0.6–1.1)	<0.001	+	Only heifer calves
[127]	Sweden	Observation	3081/122	Dairy	Diarrhea (90 d)	Parity	−	−	−	=	Only heifer calves
[128]	USA	Observation	449/3	Holstein	Diarrhea (90 d)	Parity	−	−	−	=	−
[129]	Netherlands	Observation	683/13	Holstein	Omphalitis	Parity = 2	Parity = 3	−	0.02	+	−

* No definition of time at risk; OR = Odds ratio.

**Table 7:** Studies investigating the effect of twin pregnancy on calf morbidity and mortality. The reported direction of the statistically significant effect, with “+” indicating the effect was interpreted as positive or desirable, “=” as no effect or neutral effect, and “−” as a negative or undesirable effect.

Author	Country	Study Design	Animals/Herds	Breed	Outcome	Study Group	Control Group	Effect Estimate (95% CI)	*p*	Result	Comment
[114]	Iran	Observation	104,572/16	Holstein	Perinatal mortality *	Twin pregnancy	Singelton calf	OR 7.58 (6.92–8.29)	<0.001	−	−
[133]	USA	Observation	1905/3	Holstein	Perinatal mortality *	Twin pregnancy	Singelton calf	−	<0.01	−	Primiparous
[124]	Iran	Observation	1,163,594/2,552	Holstein	Perinatal mortality *	Twin pregnancy	Singelton calf	OR 5.62 (4.82–6.35)	<0.001	−	−
[21]	USA	Observation	1,164,233/4103	Holstein	Perinatal mortality (24 h after calving)	Twin pregnancy	Singelton calf	OR 6.5	<0.01	−	−
[115]	USA	Observation	7788/3	Holstein	Perinatal mortality (24 h after calving)	Twin pregnancy	Singelton calf	OR 2.7 (1.9–3.7)	<0.05	−	−
[116]	Ireland	Observation	304,531/−	Holstein	Perinatal mortality (24 h after calving)	Twin pregnancy	Singelton calf	OR 13.36 (11.03–16.21)	<0.05	−	Primiparous
[116]	Ireland	Observation	304,531/−	Holstein	Perinatal mortality (24 h after calving)	Twin pregnancy	Singelton calf	OR 5.95–9.6 (4.7–11.38)	<0.05	−	Multiparous
[117]	England	Observation	1097/19	Holstein	Perinatal mortality (24 h after calving)	Twin pregnancy	Singelton calf	OR 2 (1–6.1)	<0.05	−	−
[118]	Norway	Observation	246,156/14,423	Dairy	Perinatal mortality (24 h after calving)	Twin pregnancy	Singelton calf	OR 4.2 (3.8–4.8)	<0.05	−	−
[20]	Germany	Observation	13,158/46	Dairy	Perinatal mortality (24 h after calving)	Twin pregnancy	Singelton calf	OR 1.683 (1.161–2.441)	0.006	−	−
[119]	Japan	Observation	1,281,737/5,172	Dairy	Perinatal mortality (24 h after calving)	Twin pregnancy	Singelton calf	OR 9.33 (9.11–9.56)	<0.01	−	−
[85]	New Zealand	Observation	2384/1	Dairy	Perinatal mortality (48 h after calving)	Twin pregnancy	Singelton calf	OR 11.9 (5.72–24.67)	<0.05	−	−
[122]	Iran	Observation	12,283/1	Holstein	Perinatal mortality (48 h after calving)	Twin pregnancy	Singelton calf	OR 0.98 (0.6–1.6)	0.97	=	−
[134]	Ireland	Observation	11,256,112/29,299	Dairy	Perinatal mortality (48 h after calving)	Twin pregnancy	Singelton calf	OR 1.96 (1.74–2.21)	<0.001	−	−
[87]	Iran	Observation	14,546/3	Holstein	Perinatal mortality (48 h after calving)	Twin pregnancy	Singelton calf	OR 3.39 (2.85–3.55)	<0.05	−	−
[118]	Norway	Observation	246,156/14,423	Dairy	Mortality first week of life	Twin pregnancy	Singelton calf	OR 1.3 (1.2–1.5)	<0.05	−	−
[102]	USA	Observation	11,465/5	Dairy	Mortality till weaning	Twin pregnancy	Singelton calf	OR 1.688 (1.105–2.578)	0.015	−	−
[126]	England	Observation	392/7	Holstein	Total protein calf plasma (1–7 d after birth)	Twin pregnancy	Singelton calf	−	−	=	−
[135]	Czech Republic	Observation	1175/33	Dairy	FTP	Twin pregnancy	Singelton calf	−	0.28	=	−
[36]	Mexico	Observation	4409/1	Holstein	Adequate transfer of passive immunity	Singleton calf	Twin pregnancy	OR 1.4 (1.1 –1.8)	0.0074	+	−

* no definition of time at risk; OR = Odds ratio; FTP = Failure of passive transfer.

## Data Availability

All data used in this study are included in the article.

## Abbreviations

The following abbreviations are used in this manuscript:

THI	Temperature–humidity index
IgG	Immunoglobulin G
BCS	Body condition score
BRD	Bovine respiratory disease
OR	Odds ratio
DCAD	Dietary cation-anion difference
HR	Hazards ratio
FTP	Failure of passive transfer
IRR	Incidence rate ratio
