# Correction: Trzebiatowski et al. Prenatal Factors Influencing Calf Morbidity and Mortality in Dairy Cattle: A Systematic Review of the Literature (2000–2024). *Animals* 2025, *15*, 1772

**DOI:** 10.3390/ani15202948

**Published:** 2025-10-11

**Authors:** Lukas Trzebiatowski, Frederike Wehrle, Markus Freick, Karsten Donat, Axel Wehrend

**Affiliations:** 1 Veterinary Clinic for Reproductive Medicine and Neonatology, Justus-Liebig University Giessen, 35392 Giessen, Germany; kdonat@thtsk.de (K.D.); axel.wehrend@vetmed.uni-giessen.de (A.W.); 2 ZAFT e.V., Centre for Applied Research and Technology, HTW Dresden—University of Applied Sciences, 01069 Dresden, Germany; frederike.wehrle@gmx.de; 3 Institute of Agricultural and Nutritional Sciences, Martin Luther University Halle-Wittenberg, 06120 Halle (Saale), Germany; markus.freick@landw.uni-halle.de; 4 Thuringian Animal Disease Fund (Institution by Law, Animal Health Service, Thüringer Tierseuchenkasse AdöR), 07745 Jena, Germany

## Error in Table

In the original publication [1], there was a mistake in Table 3 as published. When transferring data from the excel sheet to the manuscript, the addition of zinc–methionine and chrome–methionine was lost, and only methionine was reported. However, the addition of trace minerals is important for the interpretation of the results. The corrected version of Table 3 appears below.

## Text Correction

There was an error in the original publication [1]. The effects of zinc–methionine and chrome–methionine were only mentioned as effects of methionine.

A correction has been made to the following sections: 3. Results and Discussion, 3.2. Nutrition of the Pregnant Cow, 3.2.2. Study Characteristics, Paragraphs 2 and 5.

The nutrition of dairy cows in mid-pregnancy is well known to play an important role in health and performance during pregnancy and in the subsequent lactation period, which is why various feeding concepts have been developed for this phase [75]. One part of the included studies focused on the effect of adding to the diet provitamins [54–56], rumen-protected essential amino acids [59–61], rumen-protected protein [62], betaine [63], choline [64], fat [65], essential fatty acids [66–68], zinc [57], chromium [58], magnesium butyrate [69], and selenium [70] on calf morbidity and mortality. The other part of the studies investigated the influence of diets negative in dietary cation–anion difference (DCAD) [71–73] and maternal energy supply [74] on calf health.

Studies that investigated the influence of nutrition in late pregnancy on immunoglobulin transfer to calves showed a significant positive effect of adding the trace element selenium to the feed for 56 days before calving compared to a control group without additives [70]. This is in line with the finding of the improved transfer of passive immunity due to supplementation of selenium to colostrum [80] and underlines the importance of an adequate provision of selenium. The addition of rumen-protected betaine (in the last 28 days of gestation) [63], soybean oil, and fish oil (in the last 21 days of gestation) [65] also led to a significantly higher immunoglobulin transfer than in control groups without additives. In a study by Wang et al. [60], the addition of rumen-protected essential amino acids methionine and/or lysine in the last 21 days before calving resulted in improved immunoglobulin transfer to the calves. Feed additives showed no effect on the transfer of immunoglobulins in calves in other studies [54–58,62,64,66–69]. Although these studies are all designed as clinical trials and therefore show a high level of evidence, there are still wide variations in the duration and quantity of supplements used. Moreover, colostrum quality differs even in animals of one breed [81]. Based on the available data, no general recommendation on nutritional supplementation can be given to improve passive transfer of immunity.

The authors state that the scientific conclusions are unaffected. This correction was approved by the Academic Editor. The original publication has also been updated.

## Figures and Tables

**Table 3:** Studies investigating the effect nutrition of the pregnant cow on calf morbidity and mortality. The reported direction of the statistically significant effect, with “+” indicating the effect was interpreted as positive or desirable, “=” as no effect or neutral effect, and “−” as a negative or undesirable effect.

Author	Country	Study Design	Animals/Herds	Breed	Outcome	Study Group	Control Group	Effect Estimate (95% CI)	*p*	Result	Comment
[54]	USA	Clinical trial	18	Holstein	IgG calf serum (24 h after birth)	β-carotene supplementation in last 28 d of gestation	no supplementation	−	0.59	=	−
[55]	USA	Clinical trial	94	Holstein	Total protein calf serum (24 h after birth)	β-carotene supplementation in last 28 d of gestation	no supplementation	−	0.63	=	−
[56]	USA	Clinical trial	36	Holstein	IgG calf serum (24 h after birth)	Nicotinic acid supplementation in last 28 d of gestation	no supplementation	−	0.86	=	−
[57]	China	Clinical trial	40	Holstein	IgG calf serum (24 h after birth)	Zn-Methionine supplementation in last 60 d of gestation	no supplementation	−	−	=	−
[58]	Türkiye	Clinical trial	45	Holstein	IgG calf serum (24 h after birth)	Cr-Methionine supplementation in last 60 d of gestation	no supplementation or injection of levamisole	−	−	=	−
[59]	USA	Clinical trial	81	Holstein	Fecal score of the calves over first 9 weeks	Methionine supplementation in last 28 d of gestation	no supplementation	−	−	=	−
[60]	China	Clinical trial	120	Holstein	IgG calf serum (24 h after birth)	Methionine and/or Lysine supplementation in last 21 d of gestation	no supplementation	−	<0.01	+	Only heifer calves
[61]	USA	Clinical trial	78	Holstein	Medication in first 8 weeks of life (Electrolytes or antibiotics)	no supplementation	Lysine supplementation in last 26 d of gestation	OR 2.8 (1.27–6.19)	0.01	−	Only bull calves
[61]	USA	Clinical trial	78	Holstein	Antibiotics first 8 weeks of life	no supplementation	Lysine supplementation in last 26 d of gestation	OR 3.69 (1.14–12.01)	0.01	−	Only bull calves
[62]	Belgium	Clinical trial	74	Holstein	IgG calf serum (72 h after birth)	Rumen-protected protein supplementation in last 45 d of gestation	no supplementation	−	−	=	−
[63]	China	Clinical trial	24	Holstein	IgG calf serum (24 h after birth)	Rumen-protected betaine supplementation in last 45 d of gestation	no supplementation	−	<0.05	+	Only heifer calves
[64]	USA	Clinical trial	111	Holstein	IgG calf serum (24–36 h after birth)	Choline supplementation in last 28 d of gestation	no supplementation	−	−	=	−
[65]	Iran	Clinical trial	120	Holstein	IgG calf serum (24 h after birth)	Soybean oil or fish oil supplementation in last 21 d of gestation	no supplementation	−	<0.01	+	−
[66]	USA	Clinical trial	78	Holstein	IgG calf serum (24 h after birth)	Essential or conjugated fatty acids supplementation in last 56 d of gestation	no supplementation	−	0.09	=	−
[67]	USA	Clinical trial	96	Holstein	IgG calf serum (24 h after birth)	Essential fatty acids supplementation in last 56 d of gestation	no supplementation	−	0.31	=	−
[68]	Germany	Clinical trial	21	Holstein	Total protein calf serum (24 h after birth)	Conjugated linoleic acids supplementation in last 21 d of gestation	Fat supplementation	−	−	=	−
[69]	Hungary	Clinical trial	219	Holstein	Calf vitality at birth	Magnesium butyrate supplementation in last 23 d of gestation	no supplementation	−	0.001	+	−
[69]	Hungary	Clinical trial	219	Holstein	IgG calf serum; perinatal Mortality; Morbidity	Magnesium butyrate supplementation in last 23 d of gestation	no supplementation	−	−	=	−
[70]	USA	Clinical trial	60	Holstein	IgG calf serum (48 h after birth)	Selenium yeast supplementation in last 56 d of gestation	no supplementation	−	0.03	+	−
[71]	USA	Clinical trial	132	Holstein	IgG calf serum; Morbidity; Mortality;	Feeding DCAD- in last 22 of gestation	no DCAD	−	−	=	−
[72]	USA	Clinical trial	60	Holstein	IgG calf serum; Morbidity; Mortality;	Feeding DCAD- in last 21 or 42 d of gestation	no DCAD	−	−	=	−
[73]	Iran	Clinical trial	12	Holstein	Days with abnormal fecal score	Feeding DCAD- in last 21 d of gestation	no DCAD	−	<0.01	−	Only heifer calves
[74]	Cuba	Clinical trial	260	Holstein	Diarrhea (first 90 d of life)	≤75% of energy requirement in last 90 d of gestation	≥85% of energy requirement in last 90 d of gestation	−	<0.05	−	−

DCAD = diets negative in dietary cation-anion difference, OR = Odds ratio.
